# Key fungal coinfections: epidemiology, mechanisms of pathogenesis, and beyond

**DOI:** 10.1128/mbio.00562-25

**Published:** 2025-04-02

**Authors:** Danielle L. Silva, Nalu T. A. Peres, Daniel A. Santos

**Affiliations:** 1 Microbiology Department, Institute of Biological Sciences, Universidade Federal de Minas Gerais 113014, Belo Horizonte, State of Minas Gerais, Brazil; 2 Brazilian National Institute of Science and Technology in Human Pathogenic Fungi (INCT-FUNVIR), São Paulo, Brazil; Instituto Carlos Chagas, Curitiba, Brazil

**Keywords:** coinfection, fungal disease, neglected disease, pathogens, infectious disease

## Abstract

Coinfection is defined as the occurrence of at least two genetically distinct infectious agents within the same host. Historically, fungal infections have been neglected, leading to an underestimation of their impact on public health systems. However, fungal coinfections have become increasingly prevalent, emerging as a significant global health concern. This review explores fungal coinfections commonly associated with HIV, severe acute respiratory syndrome coronavirus 2 (SARS-CoV-2), influenza, *Mycobacterium tuberculosis*, and *Pseudomonas* species. These include candidiasis, aspergillosis, paracoccidioidomycosis, cryptococcosis, histoplasmosis, pneumocystosis, sporotrichosis, and mucormycosis. We discuss the key local and systemic mechanisms that contribute to the occurrence of these coinfections. HIV infects CD4+ cells, causing systemic immunosuppression, particularly impairing the adaptive immune response. The inflammatory response to SARS-CoV-2 infection disrupts both pulmonary and systemic homeostasis, rendering individuals more vulnerable to local and disseminated fungal coinfections. Severe influenza promotes fungal coinfections by triggering the production of pro-inflammatory cytokines, which damage the epithelial–endothelial barrier and impair the recognition and phagocytosis of fungal cells. Tuberculosis can replace normal lung parenchyma with collagen tissue, leading to alterations in lung architecture, compromising its function. Interaction between *Pseudomonas* and *Aspergillus* during coinfection involves the competition for iron availability and an adaptive response to its deprivation. Therefore, the specific interactions between each underlying disease and fungal coinfections are detailed in this review. In addition, we highlight the risk factors associated with coinfections, pathophysiology, epidemiology, and the challenges of early diagnosis. Recognizing the substantial worldwide public health burden posed by fungal coinfections is crucial to improve survival rates.

## INTRODUCTION

Infectious diseases have a huge impact on the global population due to their morbidity and mortality, particularly in low- and middle-income countries (1). Historically, fungal infections have been neglected, and the significance of fungi as pathogens has long been underestimated by the public health authorities. While diseases caused by other pathogens have been acknowledged as major public health concerns for centuries, fungal infections were often considered rare or of minimal public health impact (2). However, this perspective has shifted in recent years due to the increased number of immunosuppressed individuals (3), climate change (4), and human-induced environmental disturbances (5). It is estimated that there is an annual incidence of 6.5 million invasive fungal infections and 3.8 million deaths, making them the fifth leading cause of death worldwide (6, 7). Reflecting this shift, fungal infections were recently included for the first time on the WHO List of Priority Fungal Pathogens (8); however, important fungi are still neglected, such as *Sporothrix* spp. Sporotrichosis, an implantation mycosis, is relevant under the One Health perspective (9), alongside the emergence of severe forms (10, 11) and treatment failure (12), posing a significant public health challenge.

Fungal tropism is highly variable, and a single fungal pathogen can infect multiple tissues within the same host and undergo morphological changes during infection (13, 14). In many cases, nonspecific clinical manifestations lead to a lack of suspicion of a fungal infection, with health professionals often overlooking the possibility of more than one agent as responsible for the patient’s condition (15, 16). This may incur in incorrect diagnosis, leading to the unnecessary prescription of antibacterials, which contradicts the principles of rational drug use (17, 18).

The term coinfection is referred to as simultaneous, mixed, concomitant infection, polyparasitism, multiple infections, or superinfection (the latter being defined by most authors as sequential infections). It is defined as the occurrence of at least two genetically distinct infectious agents within the same host (19, 20), including pathogens from different taxonomic groups and genetic variants of the same microorganism (19, 21, 22). Intra-host competition and the interplay with the immune system influence the virulence of the infectious agents, immunity, and pathogenesis (23). Three scenarios are possible in the context of coinfections: (i) the load of one or both infectious agents may increase, (ii) one or both may be suppressed, or (iii) one may increase while the other is suppressed (19).

This review aims to explore the most prevalent fungi associated with human coinfections, the underlying mechanisms of pathogenesis, and the key factors contributing to morbidity and mortality.

## COMMON UNDERLYING DISEASES ASSOCIATED WITH FUNGAL INFECTIONS

Certain fungal infections are more likely to occur based on the combination of underlying diseases and associated risk factors. Table 1 summarizes human fungal coinfections linked to their respective combination.

**TABLE 1:** Fungal coinfections, underlying diseases, and risk factors in humans^*a*^

Fungal infection	Underlying disease/infection (reference[s])	Risk factors associated with coinfection
Candidiasis	Tuberculosis (24, 25)	Broad-spectrum antibacterials, smoking
	COVID-19 (26, 27)	Corticosteroid, mechanical ventilation, broad-spectrum antibacterial
	HIV/AIDS (28, 29)	TCD4^+^ < 200 cells/mL
Aspergillosis	Tuberculosis (30–32)	Lung damage secondary to pulmonary tuberculosis, smoking, COPD
	HIV/AIDS (33)	TCD4^+^ < 100 cells/mL
	COVID-19 (34)	Chronic liver disease, hematologic malignancies, COPD, cerebrovascular disease, invasive mechanical ventilation, renal transplant therapy, treatment with interleukin-6 inhibitors and corticosteroids
	Influenza (35, 36)	Extremes of age (infants and elderly), chronic obstructive pulmonary disease, asthma, heart failure, diabetes, blood and metabolic disorders
	*Pseudomonas* sp. (37–39)	Structural lung diseases (COPD, cystic fibrosis); immunosuppression
Paracoccidioidomycosis	Tuberculosis (40–44)	Smoking, alcohol consumption, HIV infection, immunosuppression, malnutrition, diabetes
	HIV/AIDS (45)	Smoking, alcohol consumption, TCD4^+^ < 200 cells/mL
Cryptococcosis	HIV/AIDS (46)	TCD4^+^ < 200 cells/mL
Histoplasmosis	HIV/AIDS (47)	TCD4^+^ < 200 cells/mL
Pneumocystosis	HIV/AIDS (48)	TCD4^+^ < 200 cells/mL
Sporotrichosis	HIV/AIDS (49)	TCD4^+^ < 200 cells/mL
Mucormycosis	COVID-19 (50, 51)	Diabetes mellitus, hematologic malignancies, solid organ transplant recipients, corticosteroid therapy

^*a*^ COPD: chronic obstructive pulmonary disease. NETs: neutrophil extracellular traps. CNS: central nervous system.

Tuberculosis (TB) is a highly contagious airborne disease and one of the leading causes of death worldwide. Despite being preventable and curable, it is estimated that more than 10 million people fall ill with TB each year (52, 53). Tuberculosis is associated with a significant burden of chronic obstructive pulmonary disease (COPD), even after cure (54), favoring the development of opportunistic fungal pathogens (55).

Coronavirus disease 2019 (COVID-19) rapidly gained pandemic status, leading to alarming numbers of infections and deaths worldwide since its initial outbreak in 2019 (56, 57). Severe acute respiratory syndrome coronavirus 2 (SARS-CoV-2) infection can disrupt pulmonary and systemic homeostasis, triggering an exaggerated inflammatory response that causes tissue damage and dysbiosis, characterized by reduced microbial diversity and an increased fungal load not only in the lungs but also in other organs and tissues (58). Additionally, the management of patients often involves mechanical ventilation and central venous catheters, as well as treatments with broad-spectrum antibacterials, tocilizumab, lopinavir-ritonavir, or IFN-1β (59, 60). These factors favor fungal proliferation, increasing inflammation and the risk of secondary infections (58–60). In a study conducted by our research group (26), we observed a higher risk of death in COVID-19 patients who required mechanical ventilation and had concurrent fungal coinfections.

Human influenza viruses cause highly contagious respiratory infections that can progress to severe illness and fatal complications, particularly in high-risk groups (61, 62). It is estimated that influenza leads to up to 650,000 deaths worldwide annually (35). The pathogenesis of severe influenza is driven by exacerbated inflammatory responses (63, 64) since virus-infected epithelial cells release cytokines and chemokines, which recruit an inflammatory infiltrate of neutrophils and macrophages while activating adjacent endothelial cells. This cascade amplifies the production of pro-inflammatory cytokines, such as IL-6, IL-1β, TNFα, and CCL-2, further driving immune cell infiltration, damaging the epithelial–endothelial barrier, and inducing epithelial cell death (65). Moreover, viral infection is likely to predispose to fungal coinfections by impairing key immune defenses, including phagocytosis, recognition of fungal pathogens, and the production of neutrophil extracellular traps (NETs) and reactive oxygen species (66).

The most studied high-risk factor for fungal infections is the immunosuppression caused by HIV. Nowadays, the preferred term by the WHO for AIDS is advanced HIV disease (AHD) (67). The WHO estimates that approximately 40 million people are living with HIV, and that 630,000 people died from HIV-related causes worldwide in 2022 (68). HIV primarily infects CD4^+^ cells, particularly CD4^+^ helper T lymphocytes. As the infection progresses and leads to the death of host cells, a depletion of CD4^+^T lymphocytes occurs. Since these cells are crucial regulators of the adaptive immunity, their loss effectively weakens the immune response, advancing the infection to the AHD stage, making individuals more susceptible to other infections (69).

**Fig 1:**
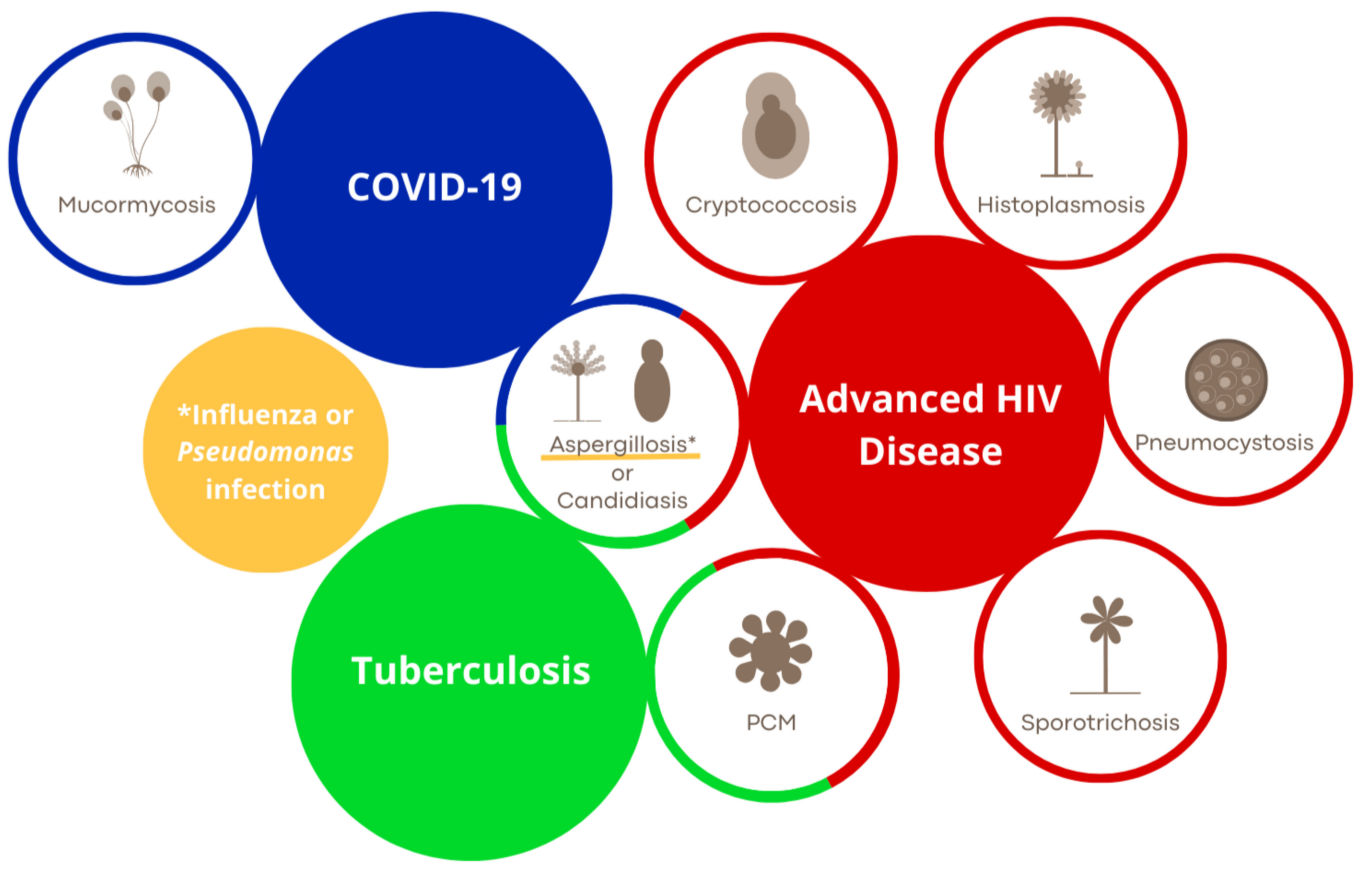
Predisposing diseases for fungal coinfections. Each underlying disease is represented by a specific color, which is also applied to the associated fungal coinfection. The intersections indicate predispositions to the respective fungal coinfection in the context of the underlying disease. In the case of aspergillosis, the asterisk (*) and specific color represent its coinfection with *Pseudomonas* and influenza. PCM: paracoccidioidomycosis. COVID-19: coronavirus disease 2019. HIV: human immunodeficiency virus.

Figure 1 summarizes the predisposing diseases for fungal coinfections.

## MOST COMMON FUNGAL COINFECTIONS

### Candidiasis

*Candida* spp. are part of the human microbiota of the skin, gastrointestinal tract, vagina, and oral cavity (70). However, under immunocompromise or dysbiosis, these yeasts proliferate excessively, causing invasive disease (71). It is estimated that 1,565,000 people develop candidemia or invasive candidiasis annually, resulting in the deaths of 63.6% of those affected (7).

As opportunistic pathogens, *Candida* spp. cause serious infections in immunocompromised patients, occurring most frequently in those admitted to ICUs (72). The estimated mortality attributed to invasive candidiasis ranges from 45% to 50% (73), but crude mortality can reach 85% in ICU patients (74). The prevalence of *Candida* species varies depending on the clinical form and the population studied. For instance, a study conducted in Chile reported a high prevalence of *Candida albicans* and *Candida tropicalis*, followed by *Candida parapsilosis* and *Candida glabrata* (*Nakaseomyces glabratus*) among hospitalized individuals. For blood and cerebrospinal fluid isolates, *C. parapsilosis* accounted for nearly half of the isolates, followed by *C. albicans* and *C. glabrata* (75). Similarly, a study in Egypt found that *C. albicans* was the most prevalent species in candidemia cases, followed by *C. tropicalis* and *C. parapsilosis* (76).

#### Candidiasis and COVID-19

*Candida* coinfections in patients with COVID-19 are referred to as COVID-19-associated candidiasis (CAC) (77). An insightful study by Kusakabe et al. (78) demonstrated that the specific immune response to yeasts correlates with COVID-19 outcomes. Significant increases in IgG antibodies specific to *Saccharomyces cerevisiae*, *C. albicans*, and *C. parapsilosis* were detected in the plasma of patients with severe COVID-19 compared with healthy controls. An increase in the abundance of *Candida* was positively correlated with disease severity (78).

Several studies emphasized the importance of intestinal mycobiota, particularly *Candida* spp., in the outcome of COVID-19. The inflammatory response to COVID-19 and the resulting intestinal and pulmonary dysbiosis play critical roles in the pathophysiology of the viral infection (79, 80). The intestinal immune system houses over 80% of the body’s total lymphocyte population. These lymphocytes induce local immune responses, and disturbances in their homeostasis can turn commensal microorganisms into pathogens (79).

The clinical relevance of candiduria in critically ill COVID-19 patients remains to be elucidated. Urinary tract infections caused by *Candida* species can arise through either the ascending or hematogenous route, potentially leading to candidemia. A study by Singulani et al. (81) found that candiduria was associated with longer hospital stays and higher mortality compared with patients without candiduria. Mediators, such as IFN-γ, IL-1ra, and CXCL-8, were significantly increased in COVID-19 patients with candiduria, with the latter two also being linked to an increased risk of death (81).

Detecting *Candida* sp. in the sputum is not often considered clinically relevant. However, studies suggested that while *Candida* spp. may not be a direct pathogen in the lungs, they could indirectly contribute to respiratory diseases. Colonization of the respiratory tract by *Candida* spp. has been associated with longer mechanical ventilation, increased ICU and hospital stays, and higher risk of death (26, 27). Similarly, isolation of *Candida* spp. from the lower respiratory tract has been linked to a worse long-term prognosis in patients with acute exacerbations of COPD (82).

In bronchoalveolar lavage samples from COVID-19 patients, *C. albicans* and *C. glabrata* are the most frequently isolated, and multiple species can be isolated from the same patient (83). In patients with candidemia, *C. albicans* is the most frequent species, followed by *C. parapsilosis*, *C. krusei*, *C. glabrata*, and *C. tropicalis* (84). An increased incidence of candidemia caused by non-*albicans Candida* species has been reported (77).

Particular concern is directed toward fluconazole-resistant *C. parapsilosis*, *C. tropicalis* (85, 86), and *C. krusei*, and multidrug-resistant strains of *C. glabrata* and *Candidozyma auris* (*Candida auris*) (87, 88). *C. auris*, which had already been emerging in different parts of the world, caused numerous cases and outbreaks in COVID-19 patients. In Brazil, the first detection occurred in a patient with COVID-19 (89), with subsequent reported cases (90, 91). Other countries have similarly reported cases and outbreaks of CAC due to *C. auris* (92–96). Unlike other *Candida* species, *C. auris* outbreaks are fundamentally nosocomial, underscoring the importance of stringent healthcare measures to prevent its spread (95).

#### Candidiasis and AHD

In addition to increasing susceptibility to exogenous coinfections, HIV also promotes changes in the commensal fungal microbiota, which can make individuals more susceptible to endogenous coinfections, such as candidiasis. It has been demonstrated that the oral mycobiome of people living with HIV/AIDS (PLWHA) differs from that of uninfected individuals (97, 98). In PLWHA, *Candida* spp. coinfection in the bronchi, trachea, lungs, or esophagus is an AIDS-defining condition (99). Oral yeast carriage and the risk of mucosal invasion increase with the progressive reduction of CD4^+^ T cells. Oropharyngeal candidiasis can occur in 80%–90% of HIV-infected individuals, and together with esophageal candidiasis, are the most common fungal coinfections in PLWHA and may represent the first sign of declining CD4^+^ T cells (28, 29).

HIV infection can also lead to intestinal barrier damage, resulting in high levels of microbial translocation. After HIV infection, CD4^+^ T-cell population in the intestine is reduced even before its reduction in blood or lymph nodes. Th1, Th17, and Th22 cells, which act in the maintenance of mucosal integrity, are decreased early. Furthermore, in the relative absence of CD4^+^ T cells, CD8^+^ T cells may be recruited to the epithelial–lamina propria interface. However, these cells become dysfunctional by expressing insufficient amounts of E-cadherin adhesion molecules. In this context of immune response failure, disruption of epithelial barrier integrity, and dysbiosis, *Candida* yeasts can proliferate and translocate into the bloodstream, with intestinal yeasts being the main source of candidemia in PLWHA (100–102). Different studies have already linked HIV infection with an increased risk of death from candidemia (103, 104). Therefore, it has been suggested that PLWHA should be monitored for the occurrence of oropharyngeal candidiasis and intestinal abundance of *Candida* spp. as a way to prevent candidemia (100, 105), and suspected cases of candidemia should be treated early to reduce mortality (104).

#### Candidiasis and tuberculosis

Pulmonary candidiasis can occur through hematogenous dissemination to the lungs or by aspiration of colonized contents from the oropharynx or stomach (106). Although *Candida* species are frequently isolated from respiratory samples, particularly from ICU patients on mechanical ventilation, interpreting its clinical significance is challenging. These findings may represent sample contamination, yeasts from the microbiota, colonization by yeasts that are not part of the microbiota but are not causing disease, or the etiological agent of a disease. Therefore, both microbiological and clinical contexts must be carefully considered to reach a diagnosis (27). Confirming *Candida* pneumonia requires evidence of tissue invasion, but obtaining a biopsy is often difficult in immunosuppressed patients. For these reasons, *Candida’s* role as a cause of lung disease remains an underestimated clinical dilemma (24).

Like *Candida* spp., *Mycobacterium tuberculosis* is a chronic colonizer of a significant portion of the human population, with approximately 30% of people worldwide being colonized by one or both microorganisms. In 90% of cases, these microorganisms coexist as commensals; however, immunological imbalances and interactions between these pathogens can lead to disease (107). For instance, *Candida* spp. secretes molecules that stimulate the growth of *M. tuberculosis*, even in mycobacteria with reduced viability or a lower multiplication rate (108). In turn, *M. tuberculosis* suppresses pyroptosis, increasing *C. albicans* burden and heightening mortality in murine coinfection (109).

The rate of *Candida* coinfection in TB patients varies widely across studies, ranging from 2.8% to 55%. Isolation rates of *Candida* species depend on the sample source. From sputum, *C. albicans* is isolated in 50% to 70% of coinfected patients. From bronchial secretions and lung tissue, *C. albicans* can be recovered in 16.5% and 20.4% of coinfected patients, respectively. *Candida tropicalis*, *C. glabrata*, and *C. parapsilosis* are the most frequently isolated non-*albicans* species (110–112).

Although the host–mycobacteria*–Candida* relationship is not yet fully understood, the potential selection of resistant yeasts from patients with multidrug-resistant tuberculosis (MDR-TB) is concerning. A study by Darma et al. (25) detected high rates of azole-resistant *Candida* in patients with MDR-TB. The study showed a dose-dependent interaction between rifampicin and fluconazole. Since rifampicin induces MDR1 expression in *C. albicans*, the authors hypothesize that chronic exposure to rifampicin in TB patients may induce azole resistance in *C. albicans*. Additionally, rifampicin increases *C. albicans* binding to fibronectin, enhancing the yeast’s adhesion, contributing to a high fungal burden in the lungs of TB patients (25, 113, 114).

### Aspergillosis

*Aspergillus* spp. are hyaline filamentous ascomycetes capable of intense sporulation (115). *Aspergillus fumigatus* is the most common etiologic agent, while *A. flavus*, *A. terreus*, and *A. niger* are less frequent (116). *Aspergillus* spp. can cause chronic non-invasive, semi-invasive, or invasive pulmonary forms of the airway tract, cutaneous, extrapulmonary, and/or disseminated forms (116, 117).

Pulmonary aspergillosis is the most common clinical manifestation. In immunocompetent individuals with no anatomical alterations in the lungs, the conidia are readily eliminated. However, in immunosuppressed individuals, the fungus can germinate and be invasive (118). Patients at greatest risk are those who had tuberculosis, sarcoidosis, bronchiectasis, cavitary neoplasia, neutropenia, and COPD (119).

Viral infections by influenza and SARS-CoV-2 may lead to severe pneumonia, creating a favorable environment for the development of aspergillosis, referred to as influenza-associated pulmonary aspergillosis (IAPA) and COVID-19-associated pulmonary aspergillosis (CAPA). Both IAPA and CAPA are associated with poor prognoses, with mortality rates reaching up to 50% despite antifungal treatment (120). The heightened susceptibility to aspergillosis induced by influenza and SARS-CoV-2 infections has been demonstrated in murine models, revealing a reduction in neutrophil recruitment to the lungs (121–123). An animal study demonstrated that protection against pulmonary aspergillosis is maintained by an axis involving innate B1a cells, natural IgG antibodies, and neutrophils (124). Viral pneumonia induces the depletion of innate B1a lymphocytes, which reduces the concentration of anti-*Aspergillus* antibodies. This impairs the formation of NETs, compromising the recognition of *Aspergillus* by neutrophils. Although NETs are not highly effective in killing *Aspergillus* spp. conidia, they exert a fungistatic effect, playing a crucial role in limiting fungal dissemination (125, 126).

Single-cell RNA sequencing (scRNA-seq) of bronchoalveolar lavage fluid from CAPA patients supports these findings, revealing significantly lower neutrophil fractions, higher NET formation, a reduced Th1 and Th17 differentiation, and transcriptional impairment of antifungal responses in CAPA patients compared with those with COVID-19 alone. Additionally, an association between reduced NET concentrations and increased mortality was observed in CAPA patients, suggesting that NET levels may hold prognostic and diagnostic values (127).

Furthermore, these patients should be monitored for clinical antifungal resistance, given the possibility of fungal resistance throughout treatment. *Aspergillus* species are intrinsically resistant to fluconazole, and some species, such as *A. terreus* may present low sensitivity to amphotericin B (128, 129). Resistance in *Aspergillus* may also result from prior exposure of the fungus to environmental fungicides, limiting therapeutic options in the clinic (130, 131).

#### Aspergillosis and COVID-19

CAPA is defined as invasive pulmonary aspergillosis occurring in temporal proximity to a previous SARS-CoV-2 infection (132). Part of the mechanism by which SARS-CoV-2 enters the cells involves the release of the fusion peptide by cleavage of the viral spike protein by a host protease. Some authors hypothesize that microbial proteases may also catalyze this reaction. In this case, *A. fumigatus* would have the potential to activate the viral spike protein, worsening COVID-19 (133).

Corticosteroid treatment does not appear to alter the alpha diversity of the lung mycobiome (134). However, it has been suggested that some therapeutic regimens to treat COVID-19 may increase the host’s susceptibility to invasive pulmonary aspergillosis (IPA). IL-6 contributes to the inflammation and severity resulting from COVID-19, and its blockade controls the cytokine storm. On the other hand, IL-6 confers a protective effect against *Aspergillus* spp., and its inhibition may increase the development of IPA (135–137).

The incidence of CAPA varied from 2.4% to 34.3% in different studies, evidencing not only natural variation but also differences in screening strategies, definitions used, and routine screening for aspergillosis in different centers (138). Furthermore, the characteristics of CAPA itself may complicate its diagnosis. These include a low rate of positive serum GM (139, 140), challenges in demonstrating angioinvasive disease (141), similarities in chest computed tomography patterns observed in COVID-19 (142), and restrictions on bronchoscopy due to the risk of SARS-CoV-2 aerosolization (143). Given these limitations, protocols provided definitions for clinical research and treatment recommendations for CAPA (132). The risk factors frequently associated with CAPA include chronic liver disease, hematologic malignancies, COPD, and cerebrovascular disease. Additionally, invasive mechanical ventilation, renal transplant therapy, and treatment of COVID-19 with interleukin-6 inhibitors and corticosteroids (34) also increase the risk of CAPA. Studies indicated that the occurrence of CAPA increases mortality and length of ICU stay (144–146). Notably, mortality was lower in patients receiving azole treatment, suggesting that initiating prophylactic antifungal therapy at ICU admission may help reduce mortality rates (134).

#### Aspergillosis and HIV

The main manifestations of aspergillosis as a coinfection in PLWHA are invasive aspergillosis (IA) and obstructive bronchial forms, although CPA and other forms may also occur (147). Aspergillosis is considered uncommon in PLWHA compared with its occurrence in people with other types of immunosuppression. However, mortality from the disease is high. In a Brazilian study, the crude mortality rates for IA and CPA were, respectively, 72.7% and 42.8% (33). In a French study, the crude mortality from IA was 68% pre-antiretroviral therapy (pre-ART), being reduced to 31% after the introduction of ART and voriconazole (148).

Although a history of tuberculosis and CD4^+^ count <100 cells/mL are among the risk factors for IA in AHD (33), depletion of CD4^+^T cells does not appear to play a central role in the pathogenesis of aspergillosis, but rather the depletion of neutrophils and macrophages (149). To combat other opportunistic coinfections, people with AHD may take medications that cause bone marrow suppression, such as sulfamethoxazole–trimethoprim and ganciclovir. The use of these medications may lead to secondary neutropenia, increasing the risk of developing aspergillosis (150, 151). Furthermore, studies indicate that aspergillosis was only diagnosed post-mortem in 21% of registered deaths (152). This finding may reflect the lack of suspicion of fungal infection and the difficulty of a proper diagnosis (153).

#### Aspergillosis and influenza

Influenza viruses are categorized into types A, B, C, and D based on genetic and antigenic differences, with types A and B being the most clinically significant for human disease (36). It is estimated that influenza causes 3 to 5 million cases of severe illness and between 290,000 and 650,000 respiratory deaths globally each year. Young children, pregnant women, the elderly, and immunocompromised individuals are particularly vulnerable to severe cases (35).

Influenza infection is a recognized risk factor for pulmonary aspergillosis, as it triggers an early exuberant influenza-induced interferon-gamma (IFN-γ) production (154, 155). The reported rates of IAPA vary across studies, influenced by the diagnostic methods used, especially those required to confirm aspergillosis. For instance, IAPA rates of 16% have been reported in the USA (156), 19% in Belgium and the Netherlands (157) and 28.1% in China (158). The occurrence of IAPA is linked to a significantly increased risk of mortality (159, 160).

Factors related to the virus, the host’s immune response, and the treatments administered can predispose individuals to IAPA. Viral infection often triggers excessive apoptosis and necroptosis of epithelial cells, leading to an amplified inflammatory response. This response damages the lung mucosa, impairs normal ciliary clearance, and increases mucus production and viscosity, therefore, predisposing to IAPA (161–163). The use of antibiotics to prevent or treat bacterial coinfections may disrupt lung microbiome, leading to dysbiosis, increasing the risk of IAPA (156, 164). The administration of corticosteroids also elevates the risk of IAPA (165).

#### Aspergillosis and tuberculosis

TB is recognized as one of the most important risk factors for chronic pulmonary aspergillosis (CPA) (30, 31, 166). However, diagnosing aspergillosis in a coinfection can be challenging, and both diseases can present clinically indistinguishable symptoms (32), often leading to aspergillosis being misdiagnosed as tuberculosis (167). Nevertheless, the global burden of aspergillosis is increasing, with approximately 2 million people suffering from CPA, half of which are related to tuberculosis (7). It is crucial that the diagnosis is confirmed, as this impacts treatment, since azoles can interact with anti-tuberculosis drugs in the serum (168).

#### Aspergillosis and *Pseudomonas*

*Pseudomonas* sp. is a motile, Gram-negative rod that thrives in diverse environments due to its remarkable metabolic versatility, biofilm-forming ability, and resistance to antibiotics (169, 170). It is a frequent infection in immunocompromised patients or with structural lung diseases, such as COPD and cystic fibrosis (CF). *Pseudomonas aeruginosa* and *A. fumigatus* are the primary pathogens infecting individuals with CF, and the prevalence of coinfection has been reported to range between 9.1% and 21.8% (37–39).

The complex interaction between these clinically relevant microorganisms has been extensively studied, revealing both reciprocal antagonism (particularly when in close proximity) and cooperation (notably when spatially distant). *P. aeruginosa* produces volatile compounds that inhibit *A. fumigatus* growth (171). The production of phenazines and pyoverdines is increased in response to the presence of the fungus (172), and pyoverdines competitively sequester iron, depriving *A. fumigatus* of this essential nutrient (173). Conversely, certain substances absent in *A. fumigatus* biofilms are produced in mixed biofilms with *P. aeruginosa*, suggesting a potential adaptive response of the fungus to the presence of the bacteria (172). However, under hypoxic conditions, such as those found in the mucus plugs of CF patients, the inhibitory effects of *P. aeruginosa* are diminished (174). Furthermore, some volatile compounds produced by *P. aeruginosa* induce the transcription of genes in *A. fumigatus* that facilitate adaptation to iron deprivation, while also creating an environment conducive to its germination in the lung (175).

It is hypothesized that mutual antagonism may enable each organism to coexist within the hostile conditions of the CF airway, maintaining equilibrium by limiting the proliferation of the other. Conversely, in a cooperative state, the enhanced virulence of these organisms could accelerate disease progression. Collectively, these findings may explain the persistence of *A. fumigatus* coinfection in CF patients harboring *P. aeruginosa* and the observed positive correlation between coinfection and an accelerated decline in lung function (175, 176).

### Paracoccidioidomycosis

*Paracoccidioides* sp. is a thermodimorphic fungus that causes paracoccidioidomycosis (PCM), an endemic infection in Latin America (177). PCM is acquired through inhalation of infectious propagules from the environment. The most common manifestation is a systemic granulomatous disease, typically involving the lungs and/or mucosa (178).

The immune response triggered by the infection plays a crucial role in determining the clinical form of the disease. A robust cellular immune response, characterized by the activation of phagocytes, is effective in controlling the infection. However, in cases of suppressed cellular immunity, such as in PLWHA, severe forms of PCM may develop (45, 179, 180).

#### Paracoccidioidomycosis and HIV

As with PCM alone, PCM-HIV coinfection occurs mainly in young men, with smoking and alcohol ingestion being risk factors for fungal disease (45). Although predominant, the chronic form appears to occur less frequently in PLWHA. The occurrence of severe and invasive PCM in PLWHA indicates an opportunistic nature of *Paracoccidioides* spp., leading to acute PCM (181), and the higher mortality rate of coinfected people compared with PCM alone. Late diagnosis may also increase the risk of complications (45). The high titer of specific antibodies against *Paracoccidioides* spp. is strongly associated with active PCM. However, since antibody production is reduced in PLWHA, the titer may fall below the detection limit, leading to false-negative results (181).

The fact that PCM is a neglected and unreported disease may underestimate its real importance. In PLWHA, the low adherence to ART contributes to the poor prognosis of PCM (182). Although uncommon, immune reconstitution inflammatory syndrome (IRIS) should be considered in the management of patients after excluding other factors that may be related to poor prognosis, like drug toxicity, antimicrobial treatment failure, and the expected course of newly diagnosed or previously diagnosed opportunistic infections (183).

#### Paracoccidioidomycosis and tuberculosis

Like PCM, risk factors for TB include those that compromise or impair the host’s immune response, with excessive and/or delayed responses against *M. tuberculosis*. These conditions include HIV infection, malnutrition, smoking, alcohol ingestion, and diabetes (40–44). The shared risk factors between both infections contribute to their simultaneous occurrence in the same patient. Indeed, coinfection with PCM and TB occurs simultaneously in up to 28% of reported cases (184–187).

PCM and TB can resemble each other in both clinical and radiographic aspects, and in a coinfection, TB is usually diagnosed before PCM, challenging the distinction between the two infections or even diagnosing both (188, 189). In these cases, the patient may present an initial response to anti-TB treatment, and the lack of a complete response may raise suspicion of fungal infection. The patient’s cure only occurs when antifungal therapy is initiated (184, 190). Incorrect and delayed treatment may increase the chances of complications, such as chronic respiratory failure (191).

### Cryptococcosis

Cryptococcosis is mainly caused by *Cryptococcus neoformans* and *Cryptococcus gattii* (192). Infection occurs through inhalation of encapsulated yeasts, which are then deposited in the pulmonary alveoli. When the immune response fails in killing the fungus, as may occur in PLWHA, it can disseminate to other anatomical sites. *C. neoformans* has a particular affinity for the central nervous system (CNS) and can cross the blood–brain barrier, leading to cryptococcal meningitis (193, 194), while meningitis due to *C. gattii* is less detected in PLWHA (195). Data on experimental coinfection highlights the importance of more studies about *Cryptococcus*–bacteria–virus coinfections in humans and a surveillance of coinfection in immunocompromised patients (196–198).

#### Cryptococcosis and HIV

*C. neoformans* was identified as a cause of human disease in the late 19th century (199). However, the incidence of cryptococcosis increased significantly during the emergence of AHD (200). ART implementation in the mid-1990s led to a dramatic decline in the rate of opportunistic infections associated with AHD, including cryptococcosis (201). Despite this, cryptococcal meningitis remains a major cause of death among HIV-infected adults, contributing to 15%–20% of global AHD-related deaths (202). In these individuals, CD4^+^ T cell count below 100 cells/µL is a risk factor for cryptococcosis (46).

Otherwise, after initiating ART, patients may develop cryptococcosis-associated immune reconstitution inflammatory syndrome (C-IRIS). This condition involves an exaggerated and dysregulated pro-inflammatory immune response that occurs with the reduction of HIV load in the peripheral blood and the beginning of CD4^+^ T cell recovery (203). Clinical manifestations of C-IRIS can be diverse and include complications related to CNS disease. Non-CNS manifestations may include fever, ocular disease, suppurative soft tissue lesions, hypercalcemia, and cavitating or nodular lung lesions, as well as lymphadenopathy, pneumonitis, multifocal disease, and soft tissue disease (204). Initiating ART during moderate immunosuppression and before significant loss of CD4^+^ T cells can significantly reduce the mortality associated with C-IRIS (203).

Delayed diagnosis, due to limitations in diagnostic techniques and lack of suspicion for cryptococcosis, is a major factor contributing to the high mortality rate associated with cryptococcal meningitis. Additionally, limited availability of antifungal medications, challenges in providing recommended intensive care, and difficulties in monitoring and managing antifungal toxicity and increased intracranial pressure further exacerbate the problem (205).

### Histoplasmosis

Histoplasmosis is caused by the dimorphic fungus *Histoplasma* spp., considered a complex of cryptic species that correlate with specific geographic distribution (206). The infection occurs through the inhalation of macroconidia and microconidia produced by the fungus. Once in the host, the fungus can be eliminated by the immune system or converted to the yeast form. This form is a facultative intracellular parasite that can survive and replicate within macrophages and other phagocytes and can cause pulmonary or disseminated disease (207). Macrophages initially act as a protected environment in which *Histoplasma* spp. can multiply and spread to other organs. After induction of cell-mediated immunity, cytokines activate macrophages to promote the destruction of yeasts (208). Histoplasmosis is one of the most common systemic infections worldwide. Its incidence ranges from 0.1 to 1 case per 100,000 population per year in temperate climates, from 10 to 100 cases per 100,000 in humid tropics, and reaches more than 100 cases per 100,000 during epidemics or in high-risk groups, including PLWHA (209). Since it is a neglected infection, reliable data on the disease and coinfections are imprecise.

#### Histoplasmosis and HIV

In Latin America, it is estimated that the incidence of histoplasmosis in some areas is higher than 1.5 cases per 100 PLWHA (210). In endemic areas, histoplasmosis may represent the first defining infection of AHD, occurring in up to 50%–75% of PLWHA (47). Simultaneous infection with different *Histoplasma* genotypes and mating types has been documented in PLWHA (21, 22). Whether this arises from multiple exposure events or from the coexistence of diverse genotypes within shared ecological niches remains uncertain. However, it is hypothesized that genetically heterogeneous infections may facilitate immune evasion and elevate the risk of disseminated histoplasmosis (22).

In PLWHA, macrophages become incapable of coordinating an effective immune response, and there is a correlation between CD4^+^ T cell count and the ability of macrophages to uptake *Histoplasma* spp. yeasts. Impaired cellular immunity leads to massive influx of macrophages with scattered lymphocytes in infected tissues, instead of well-circumscribed granulomas (211, 212). Considered primarily a pulmonary disease, histoplasmosis can progress to disseminated disease, which occurs in up to 33% in AHD patients (213–216).

### Pneumocystosis

*Pneumocystis jirovecii* is a non-cultivable Ascomycota found worldwide that can transiently colonize human pulmonary alveoli and cause severe opportunistic infections in immunocompromised individuals (217). T lymphocytes play a crucial role against *P. jirovecii* pneumonia (PJP) (218), and primary immunodeficiency or medical immunosuppression, like solid organ and hematopoietic stem cell transplantation, cancer treatments, or prolonged corticosteroid use, represent high risk for severe PJP (219).

#### Pneumocystosis and HIV

PLWHA, particularly those with low CD4^+^ cell count, are at increased risk for PJP (48). Acute PJP is more frequent in immunocompromised patients not infected with HIV compared with PLWHA (220, 221). In 1987, the year AZT was approved, it was estimated that 61% of individuals with AHD developed PJP (222). Since the introduction of ART, the incidence of PJP in this population has decreased by 21.5% (223). Despite this decline, a study conducted in Germany in 2019 indicated that HIV infection was detected in 17.1% of patients with PJP (221). Prophylaxis against PJP typically involves the use of trimethoprim and sulfamethoxazole, which inhibit dihydrofolate reductase and dihydropteroate synthase, respectively. Mutations in the genes encoding these enzymes may lead to prophylaxis failure and higher mortality rates (224, 225).

### Sporotrichosis

Sporotrichosis is a fungal infection endemic to tropical and subtropical regions and is caused by *Sporothrix* spp. (226). Typically, the infection results from traumatic inoculation of the fungus, which is commonly found in organic matter, soil, and plants. The infection spreads through the lymphatic vessels, most often leading to the lymphocutaneous clinical form. Individuals involved in activities, such as agriculture, manual woodworking, and floriculture, are at higher risk of acquiring sporotrichosis through this route (227). Zoonotic transmission, particularly through felines, is also a significant concern. Infected felines can spread the fungus through bites, scratches, or contact with their secretions, and they can infect other felines and humans (228). Less commonly, the infection may occur through inhalation (229), and in some cases, it can disseminate to other organs and systems (230).

#### Sporotrichosis and HIV

Unlike the general population, in which lymphocutaneous sporotrichosis is most common, PLWHA frequently experiences disseminated forms, including extracutaneous and cutaneous forms, accounting for up to 89.19% of cases (231), and a mortality rate of up to 43% (229). In PLWHA, a CD4^+^ cell count below 200 cells/mm³ is strongly associated with severe forms and disseminated sporotrichosis (49). A study conducted in Brazil showed that all PLWHA with sporotrichosis developed disseminated forms of the disease (232), including pulmonary (229) and meningeal sporotrichosis (233).

### Mucormycosis

Mucormycosis is caused by Mucorales fungi, mainly from the genera *Rhizopus, Mucor, Rhizomucor*, *Apophysomyces*, *Cunninghamella*, and *Lichtheimia* (234). Infection is acquired predominantly by inhalation of sporangiospores (235) and through ingestion of contaminated food (236) or by traumatic inoculation (237). These spores germinate into hyphae, which give mucormycosis a characteristic of angioinvasion with subsequent thrombosis, potential for hematogenous spread, and multiple organ involvement (50). Mononuclear and polymorphonuclear phagocytes are capable of inhibiting spore germination in healthy humans. Therefore, patients with diabetes mellitus, hematologic malignancies, solid organ transplant recipients, and those receiving corticosteroid therapy are more susceptible to mucormycosis (50, 51). In patients with ketoacidosis, the decrease in blood pH reduces the affinity of transferrin for iron, increasing free serum iron levels. This dysfunction in the regulation of glucose and iron metabolism results in reduced phagocytic capacity and intracellular killing of fungi that cause mucormycosis (238).

#### Mucormycosis and COVID-19

Mucormycosis has a global incidence of 0.005 to 1.7 per million population (239). During the COVID-19 pandemic, some countries reported cases of COVID-19-associated mucormycosis (CAM) (240–242). However, India, which already had the highest prevalence of mucormycosis in the world, experienced an unusual increase in CAM cases, with a prevalence 70 times higher (243, 244).

The most common clinical form of CAM is rhino-orbito-cerebral mucormycosis (ROCM), followed by pulmonary and cutaneous forms. ROCM begins in the nasal turbinates and progresses with vascular invasion, tissue infarction, and necrosis, which may, through contiguous spread, involve the palate, orbit, and brain (245). Different factors may contribute to COVID-19 patients being coinfected. Initially, the response to the viral infection promotes endothelial damage and increased intracellular iron. This generates oxidative burst that releases iron into the systemic circulation. Free iron uptake is essential for growth and virulence of Mucorales (244, 246, 247). Furthermore, the entry and replication of SARS-CoV-2 into the host cell induce endoplasmic reticulum stress. In response to this stimulus, the endoplasmic reticulum chaperone glucose-related protein 78 (GRP78) is overexpressed and translocated to the cell surface. This protein is also used by Mucorales as a receptor for invasion of tissues and endothelial cells. With viral replication, the expression of GRP78 is also induced by increased blood glucose levels (248).

Corticosteroids can also increase blood glucose levels, becoming a risk factor for the development of CAM (248). Added to this, SARS-CoV-2 can damage pancreatic islets and beta cells, reducing insulin secretion and consequently causing hyperglycemia (249).

## FINAL CONSIDERATIONS

Fungal coinfections involve different underlying diseases. In immunosuppressed individuals, there is a tendency for the development of severe forms and atypical manifestations. Given that fungal infections have historically been neglected, the occurrence of these manifestations, as well as those resembling other infections (e.g., tuberculosis and aspergillosis), contribute to delays and mistakes in diagnosis. It is noteworthy that coinfections should be considered in cases of atypical clinical cases, given the difficulty in isolating pathogens. This highlights the importance of understanding these infections to reduce the negative public health impacts.
